# Supplementary figures and images for: The hourglass organization of the Caenorhabditis elegans connectome
Source: PLoS Comput Biol. 2020 Feb 6;16(2):e1007526. doi: 10.1371/journal.pcbi.1007526 (PMC7029875; doi:10.1371/journal.pcbi.1007526)

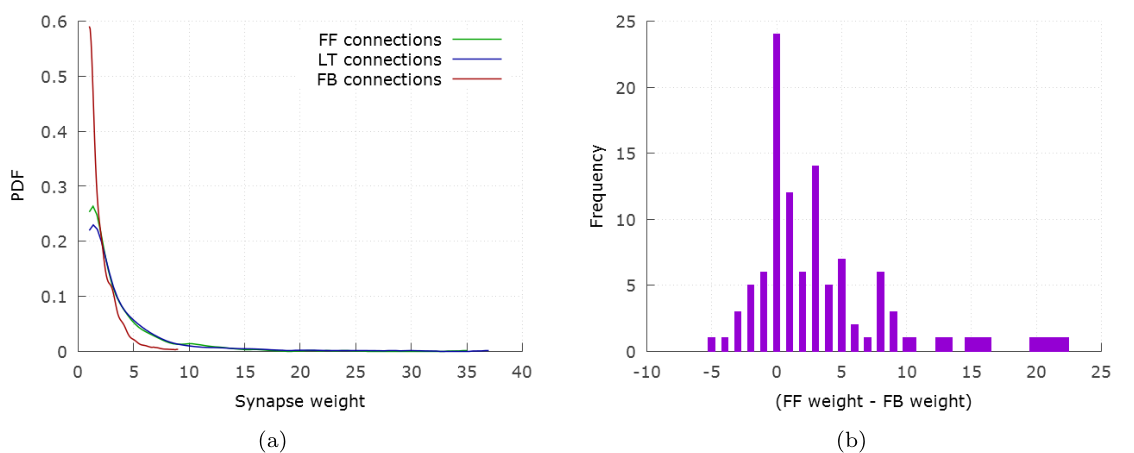

Supplement: S1 Fig — (A) Weight distribution of FF, LT and FB connections. (B) Considering only pairs of neurons with reciprocal FF and FB connections, this histogram shows the difference of the FF weight minus the FB weight. (TIFF) [file pcbi.1007526.s001.tiff]

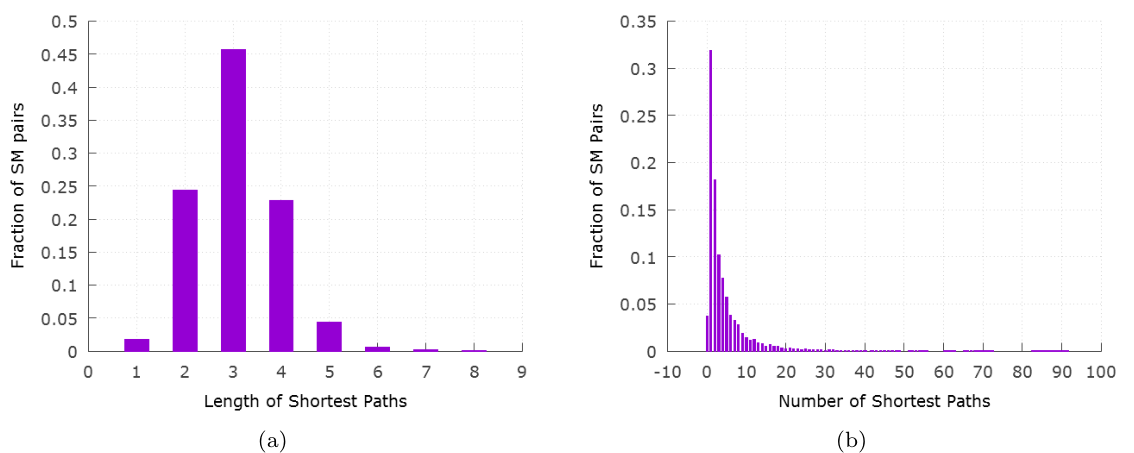

Supplement: S2 Fig — (A) The length distribution for all shortest paths from sensory to motor neurons. Almost all shortest paths are shorter than 6 hops. (B) Distribution of the number of distinct shortest paths from a sensory neuron to a motor neuron. For about 50% of S-M pairs, there are more than two shortest paths. (TIFF) [file pcbi.1007526.s002.tiff]

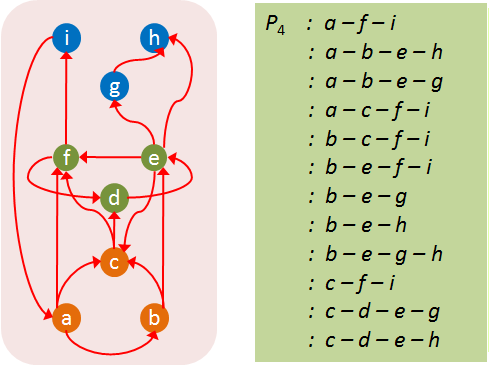

Supplement: S3 Fig — All paths for the routing scheme P4. The model network is the same one depicted in Fig 3. (TIF) [file pcbi.1007526.s003.tif]

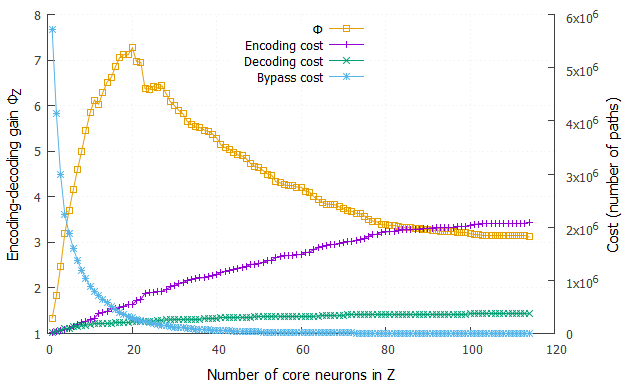

Supplement: S4 Fig — The encoder-decoder gain ratio ΦZ for the combined network containing both chemical synapses and gap junctions (contrast with Fig 12). The maximum value of ΦZ is 7.4 when Z includes the first 20 core neurons. Recall that the maximum value of ΦZ in the network of chemical synapses is 8.2. (TIF) [file pcbi.1007526.s004.tif]

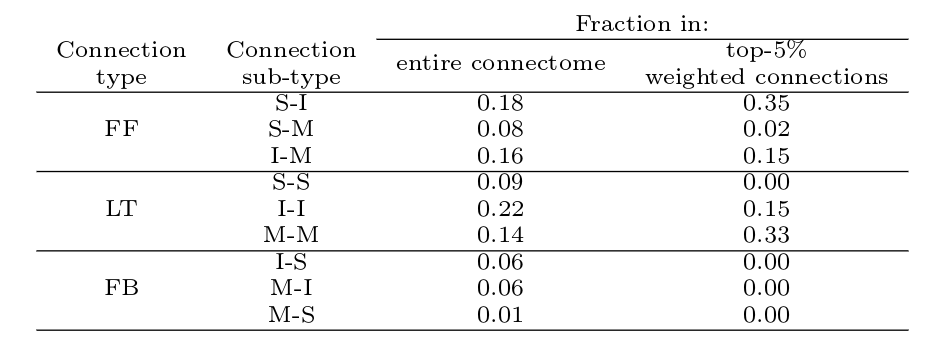

Supplement: S1 Table — In the synaptic network, the top-5% strongest connections are dominated by FF connections from S neurons to I or M neurons, and by LT connections between I neurons and M neurons. On the other hand, none of the FB connections appear in this set. (TIF) [file pcbi.1007526.s005.tif]

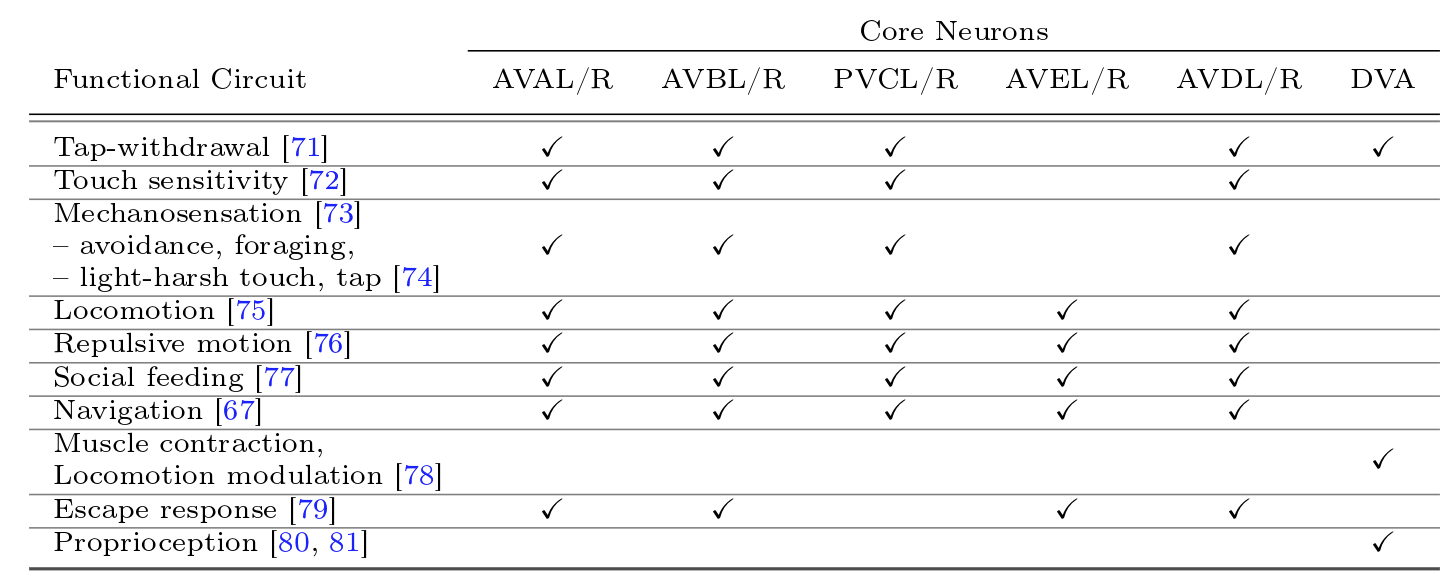

Supplement: S2 Table — Functional circuits associated with core neurons based on the C. elegans literature. The core neurons appear in several circuits, mostly related to spontaneous or planned movement. Many of the adaptive behaviors of the organism such as feeding, egg-laying, escape and navigation require a common set of underlying simpler tasks. Some of the circuits shown (e.g. thermotaxis, chemosensation, olfactory behavior) perform tasks that start with activity in some sensory neurons, followed by a locomotory response that is modulated by certain core interneurons. (TIF) [file pcbi.1007526.s006.tif]
